# Advanced Burkitt Lymphoma in Sub-Saharan Africa Pediatric Units: Results of the Third Prospective Multicenter Study of the Groupe Franco-Africain d’Oncologie Pédiatrique

**DOI:** 10.1200/JGO.19.00172

**Published:** 2019-12-03

**Authors:** Gabrielle C. Bouda, Fousseyni Traoré, Line Couitchere, Marie-Anne Raquin, Koffi M. Guedenon, Angele Pondy, Claude Moreira, Mbola Rakotomahefa, Mhamed Harif, Catherine Patte

**Affiliations:** ^1^ CHU Yalgado Ouédraogo, Ouagadougou, Burkina Faso; ^2^ CHU Gabriel Touré, Bamako, Mali; ^3^ CHU de Treichville, Abidjan, Côte d’Ivoire; ^4^ Gustave Roussy Hospital and GFAOP Database Center, Villejuif, France; ^5^ CHU Sylvanus Olympio, Lomé, Togo; ^6^ Centre Mère-Enfant, Fondation Chantal Biya, Yaoundé, Cameroun; ^7^ Hôpital Aristide Le Dantec, Dakar, Sénégal; ^8^ HJRA, Antananarivo, Madagascar; ^9^ Hôpital 20 Août 1953, Casablanca, Morocco

## Abstract

**PURPOSE:**

To evaluate the results of an intensive polychemotherapy regimen for Burkitt lymphoma (BL) in sub-Saharan African pediatric centers.

**PATIENTS AND METHODS:**

Children with advanced-stage BL (stages II bulky, III, and IV) treated with the GFAOP–Lymphomes Malins B (GFALMB) 2009 protocol in 7 centers between April 2009 and September 2015 were prospectively registered. Treatment regimen contained a prephase with cyclophosphamide followed by 2 induction courses (cyclophosphamide, vincristine, prednisone, high-dose methotrexate [HDMTX]), 2 consolidation courses (cytarabine, HDMTX), and a maintenance phase only for stage IV. HDMTX was given at the dose of 3 g/m^2^.

**RESULTS:**

Four hundred patients were analyzed: 7% had stage II bulky, 76% stage III, and 17% stage IV disease. Median age was 7.3 years, and sex ratio was 1.9:1 (male:female). A total of 221 patients received the whole protocol treatment and 195 achieved complete remission (CR), 11 of them after a second-line treatment. Treatment abandonment rate was 22%. One hundred twenty-five patients died, of whom 49 deaths were related to treatment toxicity. A total of 275 patients are alive, including 25 despite treatment abandonment, but only 110 are known to be in CR with a follow-up > 1 year, indicating a high rate of loss to follow-up. Twelve-month overall survival (OS) was 60% (95% CI, 54% to 66%) and 63%, 60%, and 31%, respectively, for stage II bulky, III, and IV. Patients with stage III disease who started second induction course within 34 days had OS of 76%, versus 57% (*P* = .0062) beyond 34 days.

**CONCLUSION:**

The GFA-LMB2009 protocol improved patients’ survival. Early dose intensity of treatment is a strong prognostic factor. Improving supportive care and decreasing loss to follow-up are crucial.

**CONTEXT**

**Key Objective**
Advanced stage Burkitt lymphomas need a short (a few months) but intensive polychemotherapy to be cured. The Groupe Franco-Africain d’Oncologie Pédiatrique developed in 7 sub-Saharan units the GFA-LMB2009, a protocol based on the Lymphomes Malins B regimen without doxorubicin. High-dose methotrexate, 3 g/m^2^, was administered with alkaline hyperhydration and folinic acid rescue 24 hours later, without methotrexate level measurements.
**Knowledge Generated**
The overall survival was 60% at 1 year (n = 400). Patients with stage III disease who started second induction course within 34 days (n = 123) had overall survival of 76%, versus 57% (*P* = .0062) for those who started beyond 34 days (n = 124). This demonstrates the importance of the early dose intensity.
**Relevance**
This study indicates that, despite many difficulties, a significant percentage of children with Burkitt lymphoma can be cured in sub-Saharan countries. It is necessary to improve supportive care and to reduce treatment abandonment and loss to follow-up.

## INTRODUCTION

Burkitt Lymphoma (BL) is the most frequent non-Hodgkin lymphoma (NHL) in children and accounts for 50%-60% of childhood NHL. It is endemic in the area known as the Burkitt belt, where it represents > 50% of childhood cancers in sub-Saharan Africa.^1-3^ BL is characterized by very rapid progression, with a tendency to early invasion of the CNS and the bone marrow (BM). More than two-thirds of the patients are diagnosed at advanced stages of the disease.^4-8^

Major advances in the management of childhood B-cell NHL were made in recent years thanks to national and international collaborations. Most of the current treatment protocols are derived from the Lymphomes Malins B (LMB) protocols designed by the Société Française d’Oncologie Pédiatrique (SFOP) or the Berlin-Frankfurt-Münster protocols of the German-Austrian group. Overall survival (OS) rate increased from 30% in the 1970s to 90% in the 1990s in developed countries through consecutive prospective studies. The identification of clinical and biologic factors and the initial response to treatment have led to stratification into therapeutic groups.^9-16^ An intensive pulse polychemotherapy of 1 to 6 months with the shortest intervals between courses and CNS prophylaxis is essential. Cyclophosphamide, high-dose methotrexate (HDMTX) and cytarabine (high dose [HD] in advanced diseases) appear as major drugs, to which are added vincristine, doxorubicin, etoposide, and corticosteroids.^3,9-17^ The improvements in supportive care and team experience led to a gradual reduction in toxic death rate from 10% to < 1% in 10 years.^9-13^ Medium- and long-term cardiac and gonadal toxicity and secondary cancer risk decreased because of all these adaptations.^9,14,16^

In developing countries, health infrastructures are insufficient and inadequate for cancer care. Access to care for the population remains limited geographically and financially, which generates diagnostic delays and abandonment.^18,19^ Moreover, human expertise is often lacking, making the application of reference protocols difficult. In the Groupe Franco-Africain d’Oncologie Pédiatrique (GFAOP), two previous studies on the treatment of BL were carried out,^4,5^ thanks to the gained experience and improved patient management of these study teams. This third multicenter study, named GFAOP–Lymphomes Malins B (GFA-LMB) 2009, was initiated in sub-Saharan units to continue to improve survival of patients with advanced-stage disease, while taking into account local conditions.

## PATIENTS AND METHODS

This is a prospective study carried out from April 2009 to September 2015. Some units joined during the study.

The protocol was reviewed and approved by the GFAOP board. Each unit was committed to present the protocol to their institution in accordance with ethics standards for research in the country.

### Eligibility

All patients ≤ 18 years old with BL were registered in the database. Children with stage III and IV disease without CNS involvement and with < 70% blasts in BM were eligible for the study. However, some patients with > 70% blasts in BM and/or CNS disease were included by medical decision. In November 2012, patients with stage II bulky disease were also included. Criteria of exclusion were: terminal disease at presentation, HIV positivity, prior chemotherapy, intensive or prolonged corticosteroid treatment, and socioeconomic conditions considered incompatible with the treatment. Families had to pay for their child’s care, except for drugs sent by GFAOP. Parents had to give consent to participate to the study.

### Diagnosis and Staging

Diagnosis was made by cytology using fine-needle aspiration of the tumor or a pathologic fluid (pleural, ascites) and/or histopathology. Immunohistochemistry was not available and not performed. Mandatory investigations included clinical examination, abdominal ultrasound and chest x-ray, CBC count, BM aspiration, CSF direct analysis, serum chemistry, and HIV serology. Hepatitis B serology was recommended. A computed tomography scan was sometimes performed. Murphy classification was used for staging classification.^20^ Stage II disease was also subdivided into nonbulky and bulky, defined as maxillofacial stage II with the presence of one of the criteria: tumor diameter > 7.5 cm, orbital lesion, > 3 affected regions, or lactate dehydrogenase greater than the twice normal value.

### Chemotherapy Regimen

**FIG 1:**
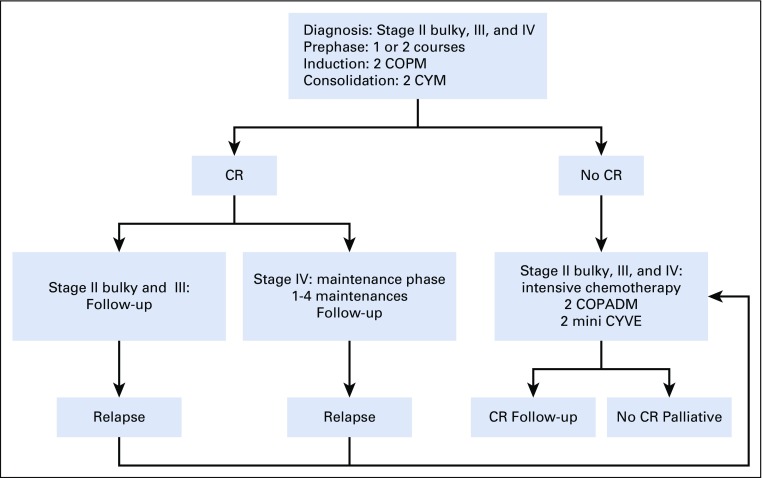
Protocol strategy. COPM, cyclophosphamide, vincristine, prednisone, high-dose methotrexate; COPADM, addition of doxorubicin (adriamycin) to COPM; CR, complete remission; CYM, cytarabine, high-dose methotrexate; CYVE, HD cytarabine, vepeside (VP16).

The GFA-LMB2009 protocol was a polychemotherapy regimen based on the LMB group B scheme without doxorubicin in the induction phase (Fig 1, Table 1).^12,16^ The prephase consisted of 1 or 2 courses of cyclophosphamide and intrathecal injection, at 1-week intervals. In the 2 induction courses of COPM (cyclophosphamide, vincristine, prednisone, HDMTX) and in the 2 consolidation courses of CYM (cytarabine, HDMTX), HDMTX was administered at 3 g/m^2^ in 3 hours followed by 12 injections of folinic acid rescue starting 24 hours later. There were 2 intrathecal injections per course. A maintenance phase with 1-4 sequences was planned for stage IV disease. In case of failure (partial remission, tumor progression, or stable tumor), more intensive chemotherapy was recommended, including doxorubicin (COPM course; including doxorubicin [adriamycin] in COPADM), HD cytarabine (CY), and etoposide (vepeside [VE] at reduce dose [mini-CYVE courses])^21^ when supportive care could be ensured.

**TABLE 1:**
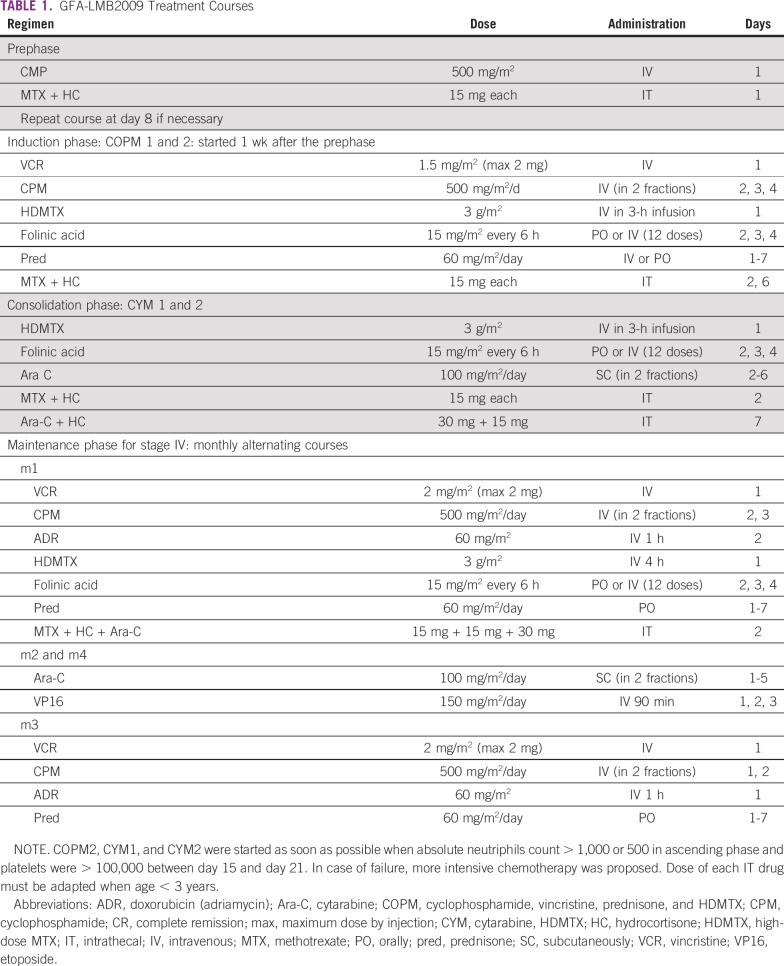
GFA-LMB2009 Treatment Courses

### Supportive Care

Patients were systematically treated for parasites according to the local ecosystem by albendazole and/or metronidazole. Recommendation for prevention/treatment of tumor lysis syndrome (TLS) was detailed in the protocol using alkaline hydration and allopurinol. Nutritional support was given, and the infected patients were treated with antibiotics and/ or antifungals according to the case. Transfusion was recommended when hemoglobin level was < 7 g/dL, or in case of clinical intolerance, and when platelet count was < 20 10^9^/L, or in case of a hemorrhagic syndrome.

### Response Criteria

Patients were evaluated clinically after each course. Complete remission (CR) had to be obtained after consolidation. CR was defined as disappearance of all tumor masses confirmed at clinical examination, x-ray, and ultrasound. In patients with initial BM or CNS involvement, examination of BM smear or CSF was performed to confirm CR. Patients in clinical CR were considered in CR even with a small residual mass stable on imaging. In case of residual masses > 5 cm, removal was recommended for histology examination to confirm remission or not.

### Statistical Method

Data were collected in each unit, recorded, transmitted, and centralized in a common database in Gustave Roussy for analysis. Overall survival (OS) was defined from the first day of chemotherapy to death or last follow-up and was estimated using the Kaplan-Meier method.^22^ Patients lost to follow-up on progression were considered as dead.

## RESULTS

Of the 562 patients registered in the database during the 6.5 years of the study, 162 were not eligible: stage IV CNS positive or with BM involvement > 70% (25%), bad nutritional status and/or socioeconomic conditions (20%), prior treatment (17%), death before treatment (15%), refusal (15%), no confirmation of the diagnosis (5%), HIV infection (3%). The 400 eligible patients were treated in 7 sub-Saharan African centers: 5 in West Africa (Abidjan, n = 71; Bamako, n = 125; Dakar, n = 15; Lomé, n = 15; Ouagadougou, n = 146), 1 in Central Africa (Yaoundé, n = 26), and 1 in Madagascar (Antananarivo, n = 2).

### Patient Characteristics

Median age was 7.3 years (8 months to 18 years) with a sex ratio of 1.9:1 (male:female). Median number of siblings was 4 (0-11). Socioeconomic level was low in 90% and middle in 10% of the families. A total of 75% of the fathers and 85% of the mothers were illiterate. Median distance to reach the unit was 185 km (1-3,000 km). The possibility of housing near the unit was mentioned by 55% of the families.

Diagnosis was based on cytology in 91% of cases, pathology in 6%, and both in 3%. Tumor sites and stages are presented in Table 2. Diagnosis of NHL without precision was reported for 25% of patients. They were included in the study on the probability of BL because of epidemiology and tumor site. BM aspiration was performed in 337 (84%) patients. Thirty-four patients had CNS involvement, and 8 had Burkitt leukemia with blasts > 70%. They should have been excluded and treated more intensively but were treated according to the GFA-LMB2009 protocol; their results are presented.

**TABLE 2:**
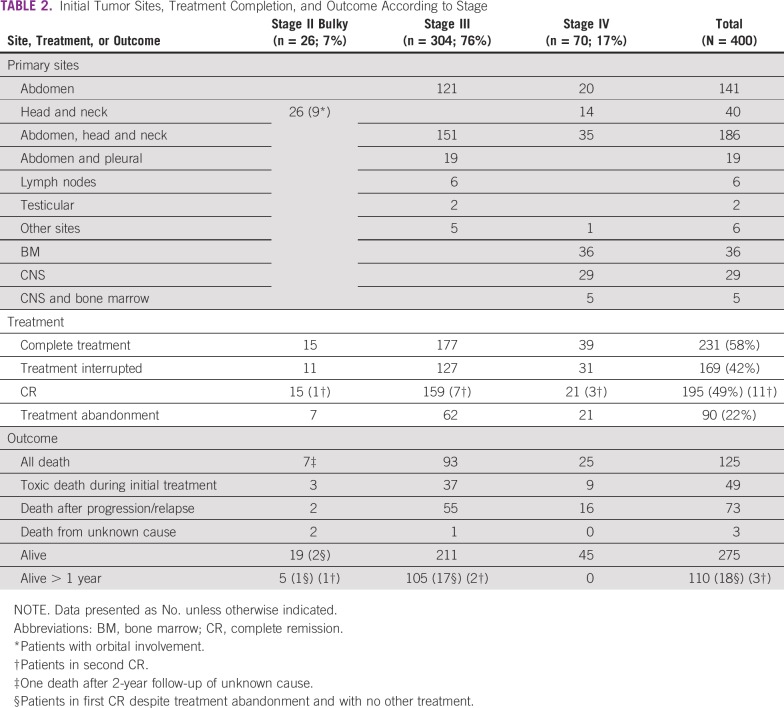
Initial Tumor Sites, Treatment Completion, and Outcome According to Stage

### Treatment and Outcome

Of the 400 patients, 231 (58%) patients received the complete treatment, whereas it was interrupted for 169 (42%; Table 2). A first complete remission (CR1) was observed in 195 patients (49%). Thirty-seven (19%) patients relapsed (32 locally and 5 in BM and/or CNS) and 31 received second-line chemotherapy, with 11 second complete remissions (CR2).

The interruption of treatment was linked to abandonment for 90 patients (22%), change for more intensive treatment because of insufficient response for 19, and deaths for 60 others. Among those who abandoned treatment, 25 patients (2 stage II, 23 stage III) were and remained in CR, and 7 patients achieved CR with another treatment (6 stage III and 1 stage IV).

A total of 125 (31%) deaths were reported. Initial treatment-related deaths included 49 patients (12%), occurring during prephase (9), COPM (29), and CYM (3) courses and treatment intensification (8); some deaths occurred while the tumor was not controlled. Seventy-three patients (18%) died as a result of a tumor and 3 from undetermined causes.

Main treatment-related toxicities of stage III are detailed in Table 3. Any grade 3 or 4 toxicity occurred in 35% of patients during the prephase, in 37% during induction phase, and in 18% during consolidation phase. Only 2 cases of TLS are reported; both patients died.

**TABLE 3:**
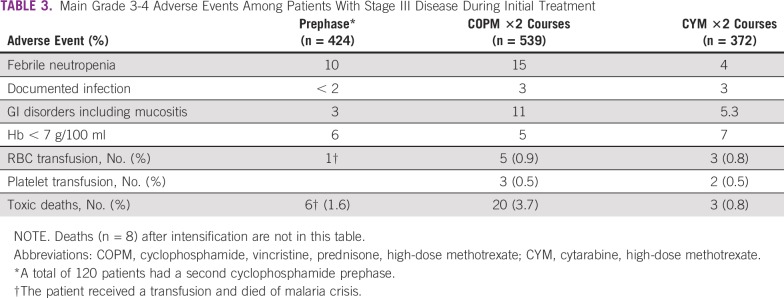
Main Grade 3-4 Adverse Events Among Patients With Stage III Disease During Initial Treatment

**FIG 2:**
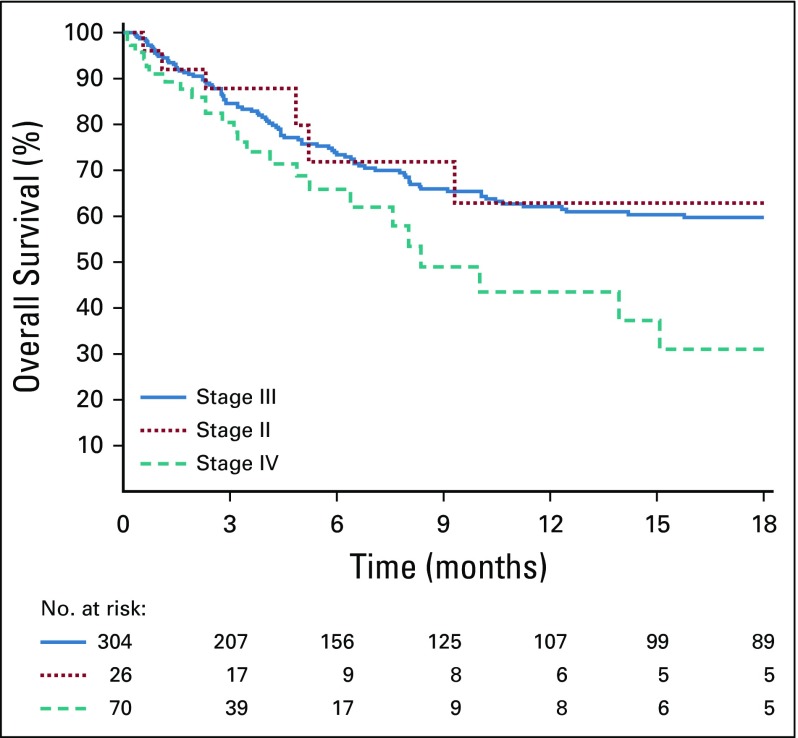
One-year overall survival of all patients according to stage.

One-year OS of all patients was 60% (95% CI, 54% to 66%) and, respectively, 63% (95% CI, 37% to 83%) in patients with stage II bulky disease, 60% (95% CI, 53% to 66%) in patients with stage III disease, and 31% (95% CI, 16% to 52%) in patients with stage IV disease (Fig 2). Among the 270 patients alive without tumor progression at last evaluation, only 110 were alive in CR with a follow-up > 1 year, including 18 who abandoned treatment.

We focused on patients with stage III disease, who represented 76% of the population. There was no statistical difference in OS whether patients were treated before or after April 2012 (*P* = .3).

**FIG 3:**
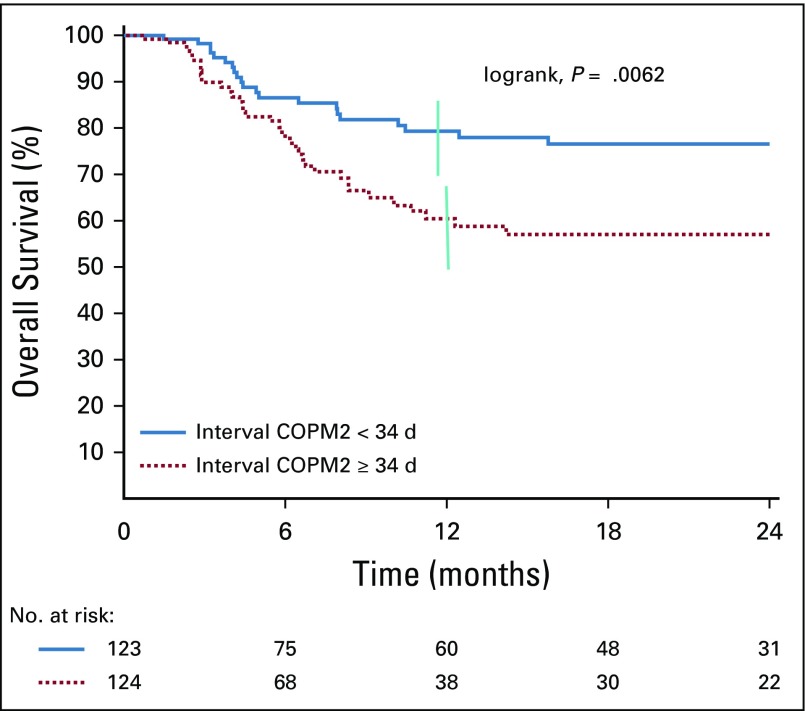
One-year overall survival of patients with stage III disease according to the interval between start of treatment and start of second induction course (COPM2). Vertical bar denote 95% CI of the of the actuarial rates.

For the 247 patients who finished induction treatment, dose intensity was measured by the interval between start of treatment and start of the second induction course (COPM2). Median interval was 34 days. OS was significantly different (*P* = .0062) if patients started COPM2 before versus later than 34 days: 76% versus 56%, respectively (Fig 3). For the 116 patients who received COPM2 after 34 days, the reason for delay > 34 days, known in 66 of 116 patients (57%), was infection or febrile neutropenia in only half of them.

## DISCUSSION

This third study of the GFAOP is the first to our knowledge that concerned 7 centers in sub-Saharan Africa and with such a high number of included patients (400). The 1-year survival rate of 60% for all the patients is encouraging, knowing that the experience of the teams was not equal in terms of seniority and that local realities were not similar, with very recent structuring of childhood cancer care in some countries.

During the 3 GFAOP studies, the number of patients treated in the sub-Saharan units increased, especially that of patients with stage III disease, who represent the majority of patients with BL (76% in the current study). In the first GFA study (2001-2004),^4^ in which most patients were from North African units, 3 sub-Saharan units enrolled 55 patients with stage III disease, whose OS was 44% (95% CI, 28% to 62%). In the second GFAOP study (2005-2009), exploring the possibility of curing patients with cyclophosphamide monotherapy,^23^ 6 sub-Saharan units participated. The 18-month OS of the 128 patients with stage III disease was only 30% using cyclophosphamide alone and 43% (95% CI, 34% to 54%) using the second-line treatment.^5^ In this third study, 304 patients with stage III disease from 7 sub-Saharan units were treated, whose survival was 60% (95% CI, 53% to 66%). Parallel to this increase in participation, it seems that survival improved, although this is difficult to demonstrate. Despite a trend toward better outcome, there was no significant difference in survival between the first and second periods of the current study (56% before April 2012 *v* 68% after; *P* = .3). This might be linked to the diverse experiences of the teams and the gradual recruitment in the study of new centers not yet accustomed to the protocol.

The association of cyclophosphamide, vincristine, prednisone, and MTX is widely used in childhood B-NHL, although the dose of each drug, especially MTX, differed among the treatment protocols. Generally in the regimen called COMP, MTX doses are between 30 mg/m^2^ and 300 mg/m^2^.^24^ In this study, GFA-LMB2009, in which courses are called COPM, MTX was administered at the dose of 3 g/m^2^ to ensure CNS prophylaxis and better systemic diffusion. This HD was feasible despite no MTX level measurement but required hyperhydration, administration of folinic acid rescue, and alkaline urine output surveillance by a trained nursing team. The common point of these protocols is the improvement of survival rates between 50% and 76.5% versus a maximum survival of 50% for monotherapies.^4,5,7,8,25-29^

The recommended interval between start of treatment and COPM2 was 25 or 32 days, depending on whether the patient received one or two prephase courses. In reality, the interval ranged from 29 to 41 days (median, 34 days). A significant difference in survival was observed for patients with stage III disease depending on the time of administration of COPM2: 76% versus 57% when administration before or after 34 days (*P* = .0062; Fig 3). This confirms the importance of early dose intensity in the treatment of BL, as was shown by the SFOP/SFCE studies, in particular in the FAB/LMB96 study.^2,16,26,30^ For the 43% of patients who received COPM2 later than 34 days, there was no significant complication reported in the previous courses. This suggests that reduction of intervals between courses could be improved by better communication between parents and trained staff, better planning of biologic examinations, and having a reserve stock of essential drugs.

Indeed, BL is the most prevalent childhood cancer in sub-Saharan Africa.^4,5,7,18,31^ It should also be recognized that drugs used for BL are among the cheapest antineoplastic drugs. However, it does not have the same consideration as malaria, tuberculosis, or HIV.^19^ Initiatives like those of the GFAOP, which aim to develop protocols adapted to local conditions, must be encouraged.^7,16,26,27^ Concerning total cost of drugs, for stage III disease the Cameroon2008 protocol^7^ is 2.5 times cheaper than the GFA-LMB2009 protocol: 166 euros versus 417 (purchasing prices by GFAOP in February 2009), with similar OS rates. This must be balanced with cyclophosphamide total dose: respectively, 7.2/9 g/m^2^ (risk 2/3: high probability of infertility) versus 3.5/4 g/m^2^ (no risk of infertility).^32,33^

Patients with stage II bulky disease were few (n = 26). Their inclusion was recent, and it was difficult to draw any conclusion.

Results in patients with stage IV disease were disappointing, with an OS of 31% and a median follow-up of only 5 months. However, it should be noted that more than half of these patients had CNS involvement (34 of 70) and/or a massive BM infiltration, which should have excluded them from the study. But the teams considered that it was not possible to give more intensive treatment similar to those that allowed an increased survival rate for patients with CNS-positive disease to 75%, while avoiding brain irradiation.^12,14,15,21^ In Davidson’s trial,^26^ survival of patients defined as group C (CNS involvement and BM infiltration > 25%) increased from 30% with COMP to 66% with LMB group C regimen (HDMTX 8 g/m^2^, and HDCY + VP16). This confirms the necessity for more intensive chemotherapy and/or other treatment, such as immunotherapy, in higher-risk BL.^26,27,34,35^

Toxic death, especially during the first month of treatment, is the main important problem in the management of BL.^7^ In the first GFAOP study with a majority of North African units, toxic death rate was 10.5%, with a decrease from 11.5% in the first year to 8% during the third year of the study, and it was 7.8% in the second study, with a less toxic regimen.^4,5^ In the current study, it was 12.25% (49 patients), with the majority of patients dying during prephase and induction (38 patients), which is a more toxic regimen performed in less-experienced sub-Saharan units. The majority of patients are admitted with advanced diseases requiring more intensive chemotherapy, and when disease is advanced the risk of toxic death is greater during the first phases of treatment.^12-14,27^ Improvements are possible in sub-Saharan units and require training of personnel at all levels. This will be obtained through organization of unit meetings with discussion on the causes of death and the way to avoid them. It is also a reason for adaptation of the treatment according to the local possibilities.^19,27,36,37^

Infections and febrile neutropenia were the main adverse effects during prephase and induction in approximately 15% of cases. Eight patients with stage III disease (2.6%) received RBC transfusions, and the estimated need was 6% on average during the treatment, indicating a need for improving the supply of blood products. Of note, these infection rates and transfusion requirements are much lower than in regimens including doxorubicin. Polychemotherapy is responsible for immunosuppression and is a source of infections requiring adapted supportive care.^4-8,12,25-27^ Only 2 cases of TLS were reported. It is possible that this adverse effect was under-reported, but prephase treatment with cyclophosphamide only, associated with TLS prevention, allowed attenuation of this manifestation and its consequences before starting induction.

Treatment abandonment rate was 22% (similar in all stages), and loss to follow-was important, since only 110 of the 275 alive patients have a follow-up > 1 year. These 2 problems still constitute major challenges for care in Africa.^18^^,^ Among the causes of abandonment, there are not only financial difficulties but also multiple social and cultural reasons that must be taken into account.^38^ However, advances are notable despite the heterogeneity of the conditions.^8^ Cancer management is costly from all aspects. Some social and psychological assistance mechanisms to help parents confronted with this hard reality are indispensable. Some reasons for abandonment can be overcome by a better involvement of all health care providers. Greater involvement of politicians and leaders is indispensable for childhood cancer care, especially in drug distribution. An improvement in pediatric oncology management will without doubt benefit all pediatric disease.

GFAOP efforts to find effective protocols with reasonable costs are in agreement with the recommendations of the Pediatric Oncology in Developing Countries Group, which has developed guidelines for the management of BL in resource-limited settings.^36^ The collaboration started in sub-Saharan Africa opens the way to extend prospective studies and research.^19,27^ In addition, biologic specificities of BL may help to initiate new therapeutic strategies.^3,39^

This trial shows that despite considerable difficulties in cancer care, especially in centers more recently opened, it is possible to cure a significant number of sub-Saharan children with BL. The GFA-LMB2009 protocol is effective for patients with stage II bulky and III BL, provided that it is applied rigorously, respecting course intervals and with improvement of supportive care.
